# Ultrafast solvent-to-solute proton transfer mediated by intermolecular coherent vibrations

**DOI:** 10.1038/s42004-026-01917-8

**Published:** 2026-01-30

**Authors:** Ramesh Jarupula, Yuezhi Mao, Haiwang Yong

**Affiliations:** 1 Department of Chemistry, University of California, San Diego, La Jolla, CA USA; 2 Department of Chemistry and Biochemistry, San Diego State University, San Diego, CA USA; 3 Program in Materials Science and Engineering, University of California, San Diego, La Jolla, CA USA

**Keywords:** Excited states, Chemical physics, Reaction kinetics and dynamics, Optical spectroscopy

## Abstract

Ultrafast photoinduced excited-state proton transfer (ESPT) plays a crucial role in protecting biomolecules and functional materials from photodamage. However, the influence of solute-solvent interactions on ESPT dynamics remains under active investigation. Here, we present an ultrafast spectroscopic study of ESPT in the photobase 2-(2´-pyridyl)benzimidazole (PBI) in methanol. Ultrafast absorption spectroscopy, supported by quantum chemical calculations, reveals three distinct kinetic steps: (1) a 2.2 ps solvent-to-solute proton transfer, (2) subsequent nonradiative relaxation to the ground state within 31 ps, producing a vibrationally hot ensemble with substantial excess kinetic energy, and (3) equilibration as this energy dissipates into the surrounding solvent bath over 186 ps. Femtosecond-resolved dynamics exhibit oscillatory signals indicative of coherent wavepacket motion on the S_1_ potential energy surface. A phase flip in the excited-state absorption maximum confirms this assignment. Fourier analysis resolves two dominant periods (∼117 fs and ∼340 fs), corresponding to in-plane and out-of-plane vibrational modes coupled between PBI and the hydrogen-bonded methanol molecule. The rapid dephasing ( < 300 fs) suggests that the nuclear wavefunction evolves on an anharmonic potential energy surface while traversing the ESPT reaction coordinate.

## Abstract (web summary)

Ultrafast photoinduced excited-state proton transfer (ESPT) plays a crucial role in protecting biomolecules and functional materials from photodamage, but how solute-solvent interactions influence ESPT dynamics is not fully understood. Here, the authors use ultrafast absorption spectroscopy supported by quantum chemical calculations to study ESPT in a photobase, 2- (2’-pyridyl)benzimidazole, in methanol, tracking the complete reaction pathway and reporting early-time intermolecular coherent vibrations that may mediate the ESPT process.

## Introduction

Excited-state proton transfer (ESPT) is a fundamental photoinduced process by which hydrogen-bonded molecules dissipate excess electronic energy via proton transfer (PT) in the excited state. Such reactions typically unfold on sub-picosecond timescales and are common in π-conjugated heterocycles, such as nucleic acid bases, where proton donation or acceptance upon excitation perturbs intrinsic acid-base equilibria^1–3^. Beyond their biological relevance, ESPT in bifunctional chromophores provides an efficient route for quenching electronic excited states, underpinning photoprotection and enabling applications in photoacids, photobases, molecular probes, photocatalysis, and fluorescence control^4^. The solvent environment is widely recognized to modulate ESPT energetics and kinetics^5^, yet how specific solute-solvent interactions shape the early femtosecond dynamics remains under active investigation.

Previous studies have illustrated the breadth of bulk solvent and vibrational motions that influence ESPT. For instance, protic solvents can form intermolecular hydrogen bonds with indigo carmine, enabling intramolecular ESPT and facilitating rapid nonradiative *S*_1_ → *S*_0_ transition^6^. Simulations of benzimidazole–phenol constructs further revealed that several coherent low-frequency vibrational modes are involved in ultrafast intramolecular proton transfer^7^. Studies on isolated molecular clusters of 2-(2´-pyridyl)benzimidazole (PBI)–water and PBI–methanol reported low ESPT barriers^8–11^, and bulk measurements have shown ultrafast intermolecular ESPT in 7-azaindole dimers and PBI in alcohols^12,13^. While coherent vibrational wavepackets accompanying intramolecular ESPT have been observed in condensed phases^14–16^, the femtosecond-resolved dynamics of solvent-to-solute proton transfer, and the potential role of coherent vibrations involving solvent molecules, remain largely unexplored. Here, we use ultrafast transient absorption (TA) spectroscopy to study PBI in methanol solution, aiming at unveiling its complete solvent-to-solute ESPT reaction pathway and exploring the vibrational coherence that accompanies the early stages of proton transfer.

PBI serves as a prototypical photobase that undergoes protonation upon photoexcitation in protic solvents. Its molecular structure contains both a basic pyridyl nitrogen and an acidic benzimidazole N–H, enabling the formation of intermolecular hydrogen bonding with solvent molecules. Gas-phase and computational studies have identified multiple solvent docking sites on PBI^8–11,17–19^, while experiments in acidic aqueous solutions have revealed ESPT leading to pyridinium tautomer formation and large-Stokes-shift fluorescence^20,21^. Furthermore, PBI’s ESPT dynamics in micellar, reverse micellar, and membrane environments have also been studied previously^22–24^, establishing it as a versatile model system for phototautomerization. Upon excitation, electron density of PBI shifts toward the pyridyl nitrogen, enhancing its proton affinity. This photobasicity gives rise to distinct photoluminescent properties and underpins emerging applications in sensing, catalysis, and optoelectronic^1^. For instance, photoexcited quinolines exhibit Brønsted/Lewis photobasicity due to charge relocalization^25,26^, highlighting the potential of photobases as light-controlled molecular switches. Within this framework, PBI functions as a photo-switchable base capable of initiating catalytic transformations without additional reagents.

In this study, we find that proton transfer occurs from a hydrogen-bonded methanol molecule to the pyridyl nitrogen of PBI within ~2.2 ps upon photoexcitation, followed by nonradiative relaxation to the ground state over ~31 ps. The excess vibrational energy released during this relaxation generates a hot ensemble that subsequently equilibrates with the solvent bath within ~190 ps. Furthermore, femtosecond absorption spectra at early time delays exhibit oscillatory signals with two distinct frequencies, closely matching low-frequency modes of the PBI–methanol complex calculated in the *S*₁ excited state. These vibrational coherences, involving both the solute and the hydrogen-bonded solvent molecule, modulate the donor–acceptor geometry and mediate ESPT dynamics immediately after excitation. The rapid dephasing of these oscillations further indicates that the nuclear wavepacket evolves away from the harmonic region toward the transition state along the ESPT reaction coordinate.

## Results

Details of both the experimental and theoretical methods used in this study are described in the “Methods” section. Briefly, we performed two sets of ultrafast TA measurements on a PBI-methanol solution upon excitation at 328 nm, focusing on different time ranges and temporal intervals. The first set of TA spectra was measured up to 600 ps, aiming to capture the complete photoinduced reaction kinetics until relaxation back to equilibrium. For the second set of TA spectra, we collected data with much finer time steps (~14 fs) up to ~1 ps, focusing on the early femtosecond dynamics accompanying ESPT immediately after photoexcitation. The temporal resolution of our instrument was determined to be ~72 fs based on fitting of the instrument response function (see SI Fig. S3). This allowed us to observe femtosecond coherent vibrations in PBI that occur right after excitation. Quantum chemical calculations were also conducted to support our experimental observations. Time-dependent density functional theory (TDDFT) at the B3LYP-D3(BJ)/def2-SVPD level was used to treat the hydrogen-bonded PBI-methanol complex (including both the PBI molecule and one methanol molecule hydrogen-bonded to the pyridyl nitrogen), while the remaining methanol solvent was treated implicitly to account for bulk solvent effects.

**Fig. 1:**
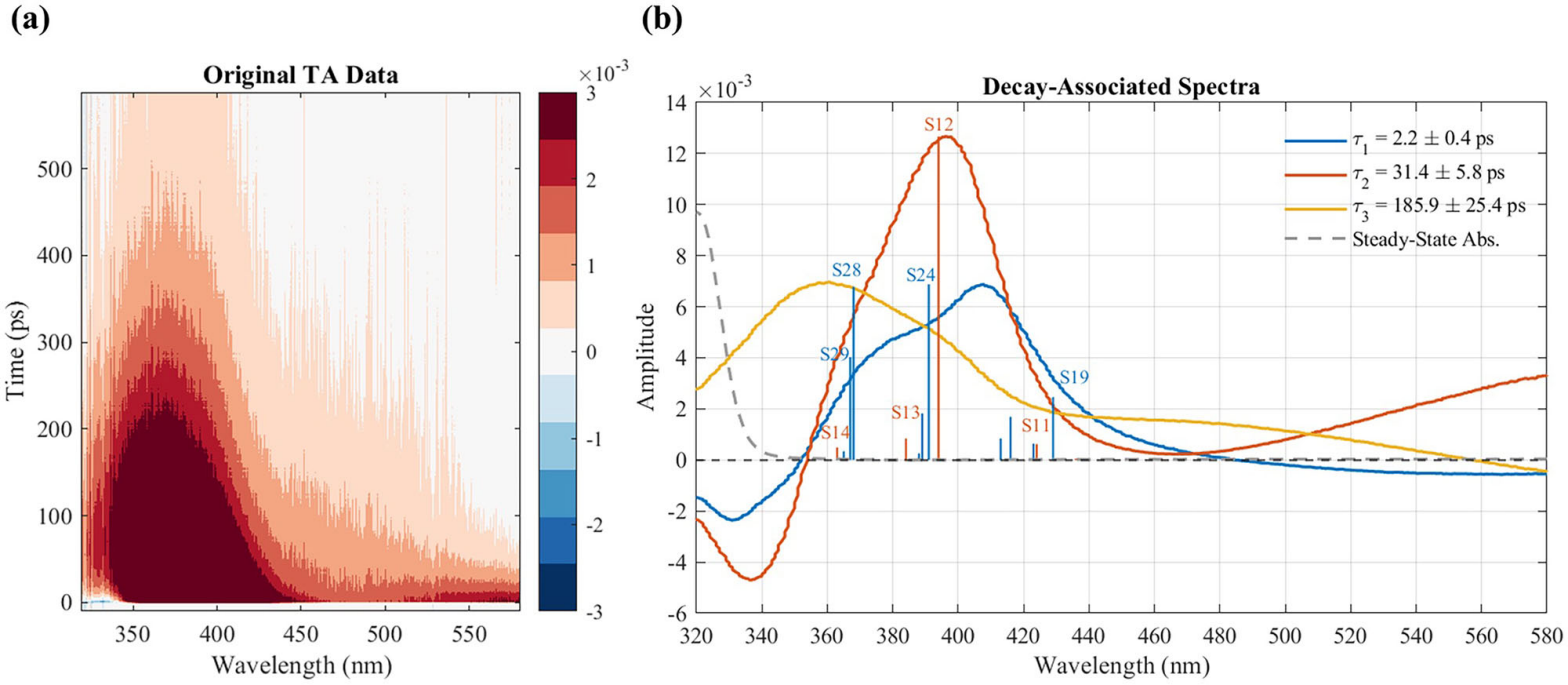
Transient absorption (TA) spectra of PBI in methanol. **a** Experimental TA spectra in OD measured up to 600 ps. **b** Decay-associated spectra from a global tri-exponential fit (*τ*_1_, *τ*_2_, *τ*_3_) of the TA data in (**a**), shown as blue, orange, and yellow curves. Lifetimes determined from the fit are provided in the legend with 1$\sigma$ uncertainties. The steady-state absorption (black dashed line) marks the ground-state bleach region. Vertical sticks, with lengths proportional to their transition dipole moment magnitudes, represent calculated S₁ → S_n_ excitations at the *S*_1_ reactant minimum (*τ*₁, blue sticks) and the *S*_1_ PT product (*τ*₂, orange sticks) geometries (see the full results of TDDFT calculations in SI Table S1).

Figure 1a displays the experimental TA spectra $\Delta A\left(t,\lambda \right)$, showing the time-dependent evolution of photoinduced kinetics over a 600 ps window. Figure 1b presents the decay-associated spectra (DAS)^27^ obtained from a global analysis of $\Delta A\left(t,\lambda \right)$ using Eq. 1, which expresses the signal as a weighted sum of exponentially decaying functions. Three exponential components were found to be sufficient to describe the data in Fig. 1a (see SI Fig. S1 for fitted spectrum and residuals), consistent with the one-dimensional fits of time-dependent signals integrated over the entire probe wavelength range (see SI Fig. S2). The global fit of Eq. 1, convoluted with the instrument response function, yields three wavelength-dependent amplitudes ${A}_{k}\left(\lambda \right)$, corresponding to three transient species with lifetimes *τ*_1_, *τ*_2_, and τ_3_.

1 $$f\left(t,\,\lambda \right)={c}_{0}+\left[{\sum }_{k=1}^{3}{A}_{k}{\left(\lambda \right)e}^{-\left(t-{t}_{0}\right)/{\tau }_{k}}\right]H\left(t-{t}_{0}\right)$$

Global analysis of the TA spectra resolves three characteristic timescales, *τ*₁ = 2.2 ps, *τ*₂ = 31 ps, and *τ*₃ = 186 ps, corresponding to distinct steps in the excited-state relaxation of the PBI-methanol complex. The DAS (Fig. 1b) shows that both *τ*₁ and *τ*₂ (blue and orange curves) retain the ground-state bleach/stimulated emission (GSB/SE) at the same spectral region close to the steady-state absorption, indicating that these species reside in the *S*₁ manifold. In contrast, the slowest component, *τ*₃ (yellow curve), lacks the GSB/SE and instead appears as a broad, red-shifted absorption, consistent with a vibrationally hot ensemble in the electronic ground state.

**Fig. 2:**
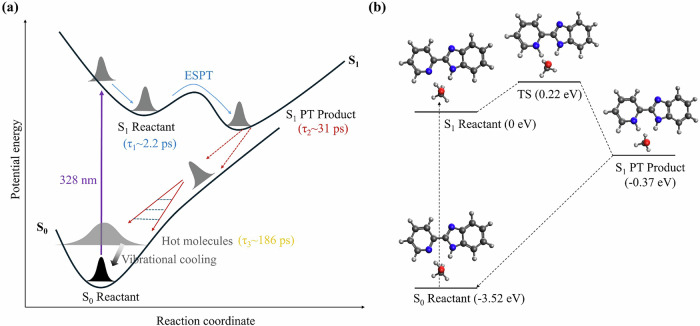
Photoinduced reaction kinetics of PBI in methanol. **a** Schematic of the reaction pathways of PBI-methanol complex upon 328 nm excitation. **b** ESPT reaction pathway calculated using TDDFT with implicit solvent, showing the *S*_0_ and *S*_1_ states near the Franck-Condon region, the *S*_1_ transition state of ESPT, and the proton-transfer product on *S*_1_. Energies are given relative to the *S*₁ reactant minimum.

Figure 2a summarizes the reaction mechanism inferred from the DAS analysis. The fastest component (*τ*₁) corresponds to the *S*_1_ reactant minimum near the Franck-Condon region, with a lifetime of 2.2 ps, undergoing ESPT to form the *S*_1_ PT product, PBIH⁺. This process involves the transfer of a proton from the hydrogen-bonded methanol to photoexcited PBI, supported by a calculated low barrier of ~0.22 eV along the proton-transfer reaction coordinate. The intermediate component (*τ*_2_) reflects subsequent relaxation of the protonated PBIH⁺ from *S*₁ to *S*_0_ via internal conversion. The resulting vibrationally hot ground-state ensemble then cools on a ~186 ps timescale as excess energy dissipates into the solvent bath.

Theoretical results corroborate these assignments: TDDFT calculations reproduce the *S*_1_ → *S*_n_ transitions of both the *S*_1_ reactant minimum and the *S*_1_ PT product, matching the excited-state absorption (ESA) region of the DAS in both peak positions and relative transition dipole moment strengths (Fig. 1b). Furthermore, comparison of theoretical and experimental results suggests that solvent-mediated hydrogen-atom transfer (HAT) in the *S*_1_ state (i.e., methanol acts as a bridge transferring a hydrogen atom of PBI from the imidazole nitrogen to the pyridyl nitrogen) is unlikely to be the dominant pathway. None of the calculated *S*_1_ → *S*_n_ transitions for the *S*_1_ HAT product match the ESA features of the DAS component associated with *τ*_2_ (see SI Table S1). This conclusion is consistent with the calculated reaction pathways of the *S*_1_ HAT (SI Fig. S4) and *S*_1_ PT (Fig. 2b), which show that the ESPT product is energetically more stable than the HAT product by ~0.1 eV.

**Fig. 3:**
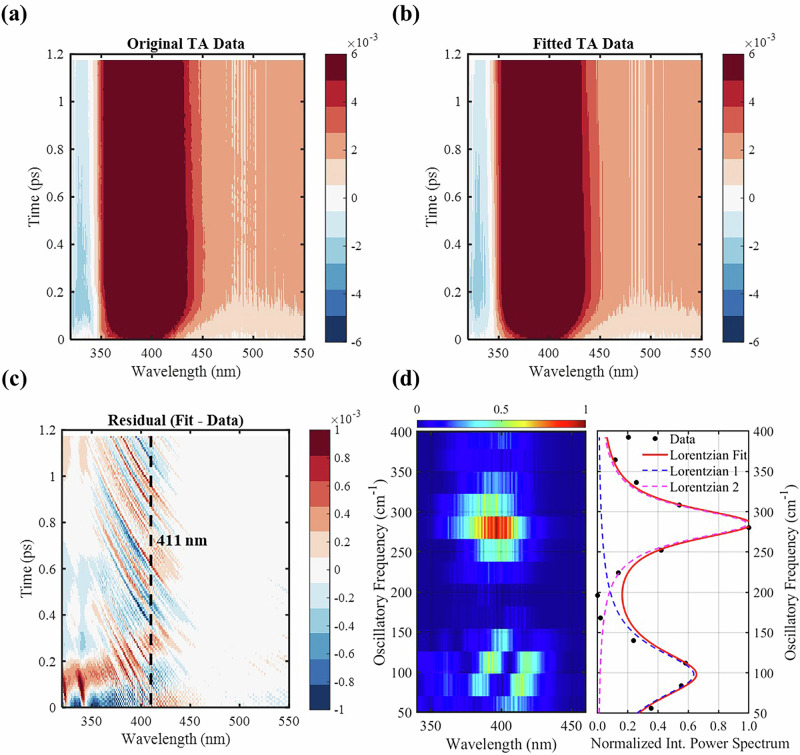
Ultrafast TA spectra with femtosecond temporal resolution, focusing on early time ranges. **a** Experimental TA spectra in OD measured with finer time steps up to 1.2 ps. **b** Fitted TA spectra of data in Fig. 3a, obtained from decay-associated spectra analysis with a single-exponential component corresponding to τ₁ in Fig. 1b. **c** Residual map obtained by subtracting the experimental data from the fitted spectra, revealing oscillatory components along time and a phase flip centered around 411 nm. **d** Two-dimensional power spectrum derived from Fourier analysis of the residual map, along with the normalized intensity profile of the power spectrum integrated over wavelengths (black dots) and its corresponding fit (red line) with two Lorentzian functions, yielding two dominant frequency components at ~98 cm⁻¹ (blue dashed line) and ~285 cm⁻¹ (magenta dashed line) with dephasing times (*τ*_d_) of 130 fs and 210 fs, respectively, as determined from the fitted Lorentzian linewidths.

While the previous DAS analysis captures the overall excited-state kinetics, it cannot fully resolve the ultrafast dynamics occurring immediately after photoexcitation. This limitation arises because the global fit assumes that each exponential component represents a well-defined transient species, neglecting coherent nuclear motions that may accompany the earliest stages of proton transfer. To probe these faster processes, we recorded TA spectra with finer temporal resolution, focusing on the early time ranges (Fig. 3a), enabling direct observation of the sub-picosecond dynamics associated with the onset of solvent-to-solute proton transfer. The DAS analysis of the early-time spectra (Fig. 3a) using a single-exponential model (Fig. 3b) reproduces the overall evolution of the signal, yielding a lifetime of 1.8 ps. This is consistent with the fastest kinetic component (*τ*₁ = 2.2 ps) determined previously. This agreement confirms that both datasets describe the same ESPT event, while the early-time dataset resolves additional features that were previously obscured.

The residual map (Fig. 3c) reveals pronounced oscillatory features along the time axis. A characteristic phase flip (i.e., phase inversion in the wavepacket) near 411 nm (dashed line) coincides with the ESA maximum band of the τ₁ component in Fig. 1b. Previous studies have established that such a phase flip at the ESA maximum indicates an excited-state wavepacket, whereas a phase flip at GSB maximum signifies ground-state wavepacket^28,29^. The absence of a comparable phase flip in the 320–350 nm region, where GSB dominates, further confirms that the observed oscillations originate from excited-state vibrational coherence. This coherence reflects early-time nuclear wavepacket motion on the *S*_1_ potential energy surface, directly coupled to the ESPT dynamics right after photoexcitation.

Fourier transformation of the oscillatory residuals yields the two-dimensional power spectrum shown in Fig. 3d, revealing two dominate frequency components centered in the ESA spectral region. Integration of the intensity over wavelength produces two distinct peaks, which can be accurately fitted (red trace) by the sum of two Lorentzian functions, yielding center frequencies of ~98 cm^−1^ (vibrational period = 340 fs) and ~285 cm^−1^ (vibrational period = 117 fs). The corresponding dephasing times, derived from the Lorentzian linewidths, are 130 fs and 210 fs, respectively. The rapid dephasing of these oscillations likely arises from intrinsic vibrational decoherence and fast intramolecular vibrational energy redistribution as the nuclear wavepacket evolves away from the harmonic region toward the barrier along the ESPT reaction coordinate.

The observed frequencies align closely with the low-frequency normal modes of the hydrogen-bonded PBI–methanol complex at the *S*_1_ reactant geometry, calculated using TDDFT with implicit solvent (B3LYP-D3(BJ)/def2-SVPD/SMD(methanol)). In particular, the 5th (102 cm^−1^) and 12th (285 cm^−1^) vibrational modes correspond closely to the experimentally determined frequencies. In addition, the 4th mode (90 cm^−1^; period = 371 fs) may also contribute, given its close match to the 340 fs period of the first oscillation. The corresponding displacement vectors (SI Fig. S5) show that: the 4th mode involves a collective in-plane bending and torsional motion of the methanol molecule relative to PBI, primarily modulating the hydrogen-bond geometry between the pyridyl nitrogen and the methanol hydroxyl group; the 5th mode exhibits a concerted twisting of the benzimidazole and pyridyl rings, coupled with a wagging of the methanol hydrogen, representing a hydrogen-bond–assisted torsion that perturbs the donor–acceptor alignment along the ESPT coordinate; and the 12th mode corresponds to a higher-frequency out-of-plane skeletal deformation of the PBI framework modulating the π-conjugated backbone. Together, these modes suggest how low-frequency nuclear motions couple solvent and solute geometries in the excited state by dynamically modulating the hydrogen-bonding and donor–acceptor orientation. Their combined activity supports the picture of a multimode vibrational coherence during the ESPT process, in which both solute and solvent motions participate. Such multimode coupling has been theoretically predicted for intramolecular ESPT systems^7^, and here our results show that such vibrational coherences can also emerge in intermolecular ESPT, where hydrogen-bonded solvent molecules play an active role.

In summary, using ultrafast transient absorption spectroscopy combined with quantum chemical calculations, we unveiled the complete reaction pathway of solvent-to-solute proton transfer in a prototypical photobase, as well as the early-time femtosecond coherent vibrations that may mediate the ESPT process. Our study presents an intriguing case of intermolecular ESPT accompanied by vibrational coherences involving multiple modes. It is worth noting that the precise manner in which these coherent vibrational modes facilitate or inhibit proton transfer remains an open question. Future studies aimed at disentangling the roles of these modes, whether they actively promote, modulate, or impede the proton-transfer process, would be highly valuable. In this regard, emerging ultrafast techniques such as solution-phase X-ray and electron diffraction have shown great promise for resolving coherent vibrational motions in solution phase with both atomic spatial and femtosecond temporal resolution^30–34^. Extending the present study using these approaches could directly reveal the underlying nuclear dynamics and help elucidate the role of vibrational coherence in solvent-mediated ESPT reactions.

## Methods

### Experimental

2-(2′-Pyridyl)benzimidazole (PBI, 97% purity) was purchased from Sigma-Aldrich and used without further purification. A 1 mM solution was prepared in HPLC-grade methanol. Ultrafast TA experiments were performed using a HELIOS pump-probe spectrometer (Ultrafast Systems). The output of a regenerative amplifier Ti:sapphire laser (Coherent Astrella, 800 nm, 35 fs pulses, 5 kHz repetition rate) was directed into an optical parametric oscillator (OPA) to generate the pump wavelength at 328 nm, which excites PBI in methanol from *S*_0_ to *S*_1_. A broadband depolarizer was inserted in the pump line to minimize anisotropic effects. The pump pulse energy was attenuated to ~100 nJ to avoid multiphoton excitation. A white-light continuum probe was generated from 10% of the 800 nm output, yielding a spectral window of 320–580 nm. The probe time delay relative to the pump pulse was controlled with an automated delay stage, providing delays from femtoseconds to several hundred picoseconds. The overall instrument response function was modelled as a Gaussian of width $\sigma =72\,\pm 1$ fs from the fitting, as shown in SI Fig. S3. Time-resolved spectra were obtained by subtracting the pump-off from the pump-on signal at each time delay, and a standard chirp correction procedure^35^ was applied to yield the final TA data in OD, $\Delta A\left(t,\lambda \right)$, as shown in Figs. 1 and 3.

### Computational

The ESPT reaction pathway in S_1_ (Fig. 2b) was calculated using time-dependent density functional theory (TDDFT)^36,37^ for the hydrogen-bonded PBI-methanol complex in implicit solvent at the B3LYP-D3(BJ)/def2-SVPD/SMD(methanol) level^38–41^ using the ORCA 6.0 software package^42^. The B3LYP functional has previously been shown to yield excitation energies and vibrational frequencies of the PBI-methanol complex in excellent agreement with experimental values measured in gas-phase clusters^9^. The Tamm-Dancoff approximation^43^ was employed as the default setting in ORCA. Structures of the *S*_1_ reactant (minimum near the Franck-Condon region) and PT product were optimized. An alternative product on the *S*_1_ surface, corresponding to a solvent-mediated hydrogen atom transfer (HAT)^9^ from the imidazole ring to the pyridyl ring, was optimized at the same level of theory. Its energy was calculated to be ~0.1 eV higher than that of the *S*_1_ PT product. The transition-state (TS) structures for PT and HAT were located using the nudge elastic band (NEB)^44^ method, followed by strict TS optimizations. The corresponding ground-state (*S*_0_) surface was also calculated, showing a monotonic increase in energy along the proton-transfer coordinate with no minimum corresponding to the PT product in *S*_0_. Harmonic frequency calculations were performed at the *S*_1_ reactant geometry in comparison with the experimental results (see SI Table S2).

To examine ground- and excited-state absorptions, over 20 excited states were calculated at the *S*_1_ reactant, *S*_1_ PT product, and *S*_1_ HAT product geometries using full TDDFT with the same functional and basis set (B3LYP-D3(BJ)/def2-SVPD). The Q-Chem 6.2 software package^45^ was used for these calculations. A linear-response conductor-like polarizable continuum model (CPCM)^46–48^ was employed to account for solvent effects; for methanol, the dielectric constant ($\varepsilon$) and optical dielectric constant (${\varepsilon }_{\infty }$) were set to 33.0 and 1.77, respectively. Energy gaps between higher excited states *S*_n_ (*n* > 1) and *S*_1_, along with the corresponding transition dipoles, were quantified to identify the most probable excited-state absorptions from S_1_.

## Supplementary information

Supplementary Information

## Data Availability

The data supporting this study are available within the main text and the Supplementary Information. All relevant files are available from the corresponding authors upon reasonable request.
