# Supplementary material for: Ultrafast solvent-to-solute proton transfer mediated by intermolecular coherent vibrations
Source: Commun Chem. 2026 Jan 30;9:111. doi: 10.1038/s42004-026-01917-8 (PMC12960664; doi:10.1038/s42004-026-01917-8)
Supplement: Supplementary file 1 — Supplementary Information [file 42004_2026_1917_MOESM1_ESM.pdf]

Supplementary Information

Ultrafast Solvent-to-Solute Proton Transfer Mediated by Intermolecular Coherent Vibrations

Ramesh Jarupula[1], Yuezhi Mao[2]* and Haiwang Yong[1,3]*

Affiliations

[1]Department of Chemistry, University of California San Diego, La Jolla, CA 92093, USA

[2]Department of Chemistry and Biochemistry, San Diego State University, San Diego, CA 92182, USA

[3]Program in Materials Science and Engineering, University of California San Diego, La Jolla, CA 92093, USA

*Correspondence to: hyong@ucsd.edu (H. Y.) and ymao2@sdsu.edu (Y. M.)

Table S1. Calculated vertical electronic excitation wavelengths and magnitudes of transition dipole moments for the $S_1 \rightarrow S_n$ transitions of the S_1 reactant, S_1 proton transfer (PT) product and S_1 hydrogen atom transfer (HAT) product using full TDDFT (B3LYP-D3(BJ)/def2-SVPD). A linear-response conductor-like polarizable continuum model was employed to account for solvent effects. The magnitudes of the transition dipole moment vectors are given in atomic units (a.u.).

Excited States	$S_1 \rightarrow S_n$ (nm) (S_1 Reactant)	Transition Dipole (S_1 Reactant)	$S_1 \rightarrow S_n$ (nm) (S_1 PT Product)	Transition Dipole (S_1 PT Product)	$S_1 \rightarrow S_n$ (nm) (S_1 HAT Product)
2	3358.6	1.0615	4040.4	0.1410	2055.7
3	1435.0	0.9015	968.9	1.7304	1112.9
4	1167.2	0.0295	806.0	0.0721	958.4
5	1067.8	0.0935	782.1	0.0178	798.1
6	962.3	0.0530	728.5	0.0675	741.5
7	815.8	1.3191	474.8	2.4758	653.1
8	771.2	4.7210	463.5	0.2721	542.0
9	705.0	0.8369	460.3	0.0022	511.5
10	602.3	0.9725	436.5	0.0049	508.5
11	594.5	0.0050	423.7	0.0539	485.0
12	567.2	0.3437	393.9	1.0965	445.0
13	530.9	0.0662	384.5	0.0724	444.3
14	522.9	0.0215	362.9	0.0427	420.4
15	482.4	0.3827	357.9	0.0272	411.4

16	465.5	0.0092	348.4	0.0077	374.5
17	461.0	0.3914	330.4	0.0459	366.0
18	440.4	0.0381	326.2	0.0418	365.7
19	429.3	0.3638	317.4	1.2629	365.5
20	424.0	0.0118	311.4	0.1407	356.7

Table S2. Calculated vibrational frequencies for the S_1 reactant geometry using TDDFT with implicit solvent (B3LYP-D3(BJ)/def2-SVPD/SMD(methanol)).

Normal Mode	Frequency (cm^{-1})	Normal Mode	Frequency (cm^{-1})
1	39.95	43	1050.47
2	59.49	44	1060.77
3	64.10	45	1103.69
4	90.28	46	1109.98
5	102.02	47	1137.56
6	118.17	48	1143.69
7	131.16	49	1161.78
8	147.02	50	1184.83
9	159.90	51	1205.83
10	220.63	52	1234.89
11	257.67	53	1264.26
12	285.36	54	1316.24
13	305.58	55	1353.27
14	342.60	56	1374.17
15	413.38	57	1381.75
16	424.95	58	1413.74
17	462.11	59	1435.51
18	511.69	60	1447.20
19	563.74	61	1451.85
20	582.81	62	1457.75
21	588.76	63	1467.39
22	638.99	64	1480.52
23	694.46	65	1499.34
24	700.43	66	1508.43
25	713.91	67	1517.00
26	729.66	68	1565.50
27	795.70	69	1584.38
28	800.60	70	1621.67
29	821.27	71	1657.05
30	835.6	72	2907.28
31	845.87	73	2993.79

32	879.66	74	3060.19
33	899.83	75	3105.86
34	920.00	76	3163.49
35	940.00	77	3167.47
36	984.55	78	3189.37
37	987.25	79	3196.84
38	991.19	80	3203.72
39	997.64	81	3211.52
40	1006.29	82	3230.63
41	1010.78	83	3241.96
42	1042.43	84	3279.44

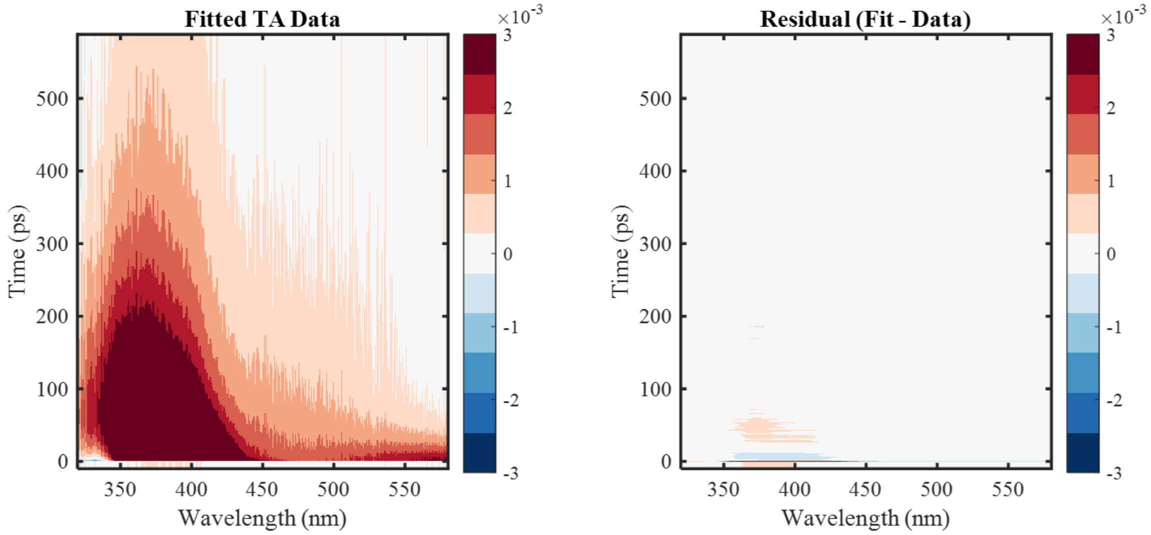

Figure S1. Fitted TA spectra of the long-range scan in Figure 1a obtained from DAS analysis with three exponential components, along with the corresponding residuals.

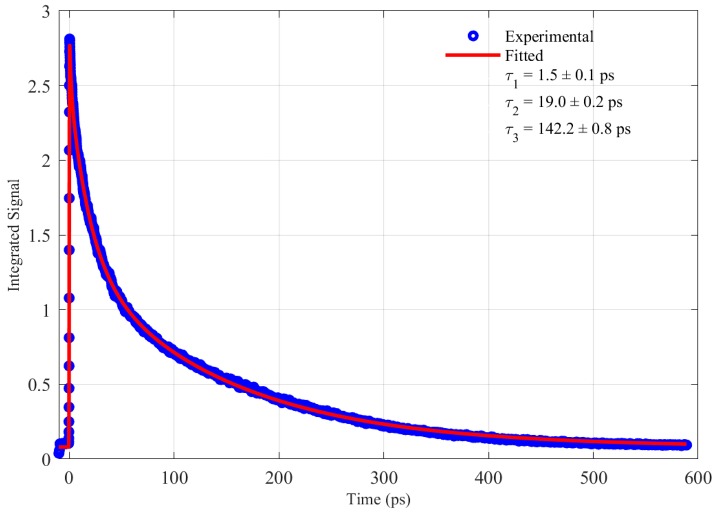

Figure S2. The experimental time-dependent TA signal (blue dots), integrated over all wavelengths from the data in Figure 1a, with fit (red curve) to a three-exponential model convoluted with the instrument response function.

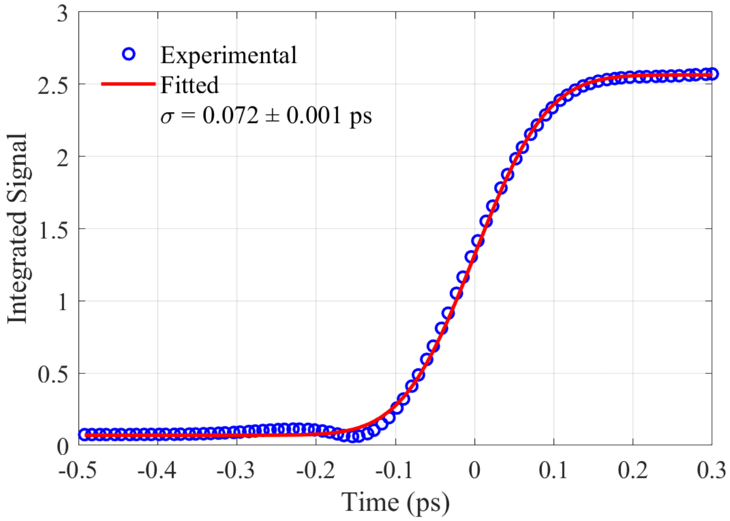

Figure S3. The measured Instrument Response Function (IRF), showing a Gaussian width of 72 fs. The fit is performed on the absolute signal integrated over all wavelength ranges from the data in Figure 3a.

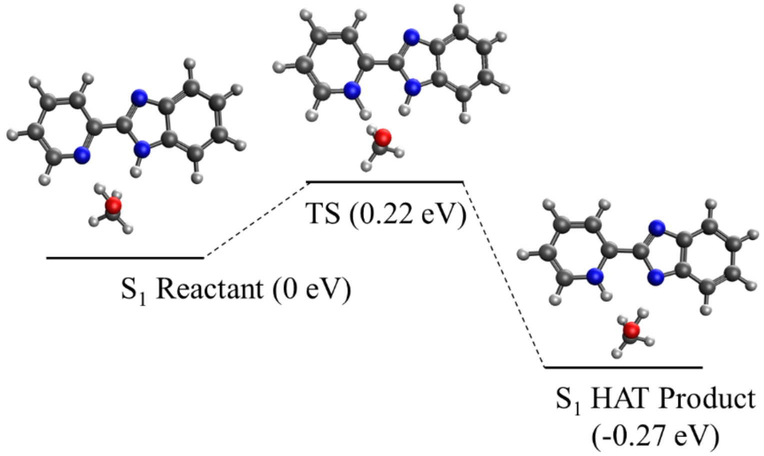

Figure S4. S_1 Hydrogen atom transfer (HAT) reaction pathway calculated using TDDFT with implicit solvent.

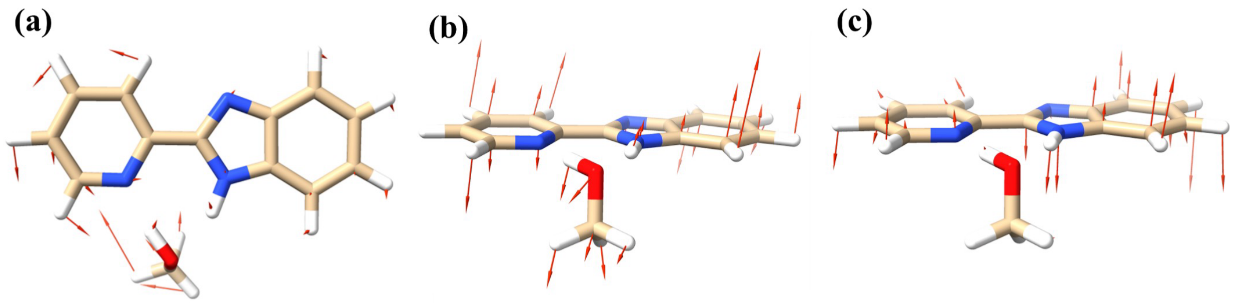

Figure S5. Displacement vectors of the calculated normal modes at (a) 90 cm^{-1}, (b) 102 cm^{-1}, and (c) 285 cm^{-1} for the PBI–methanol complex at the S_1 reactant geometry, corresponding to the 4th, 5th, and 12th modes in Table S2.

Table S3. Optimized stationary geometries of the PBI-methanol complex with implicit solvent.

S_0 minimum:

30
E = -741.861003549548 a.u.

C	-5.00318654682974	2.75619287221400	0.14832968269168
C	-4.93286901921673	1.36691115471313	-0.10934797336943
C	-3.85465190770581	3.53348897023713	0.26300860538363
C	-2.61208761477648	2.89491840455791	0.11452062024843
C	-2.55964971336934	1.49903382744822	-0.14432786947631
C	-3.71187651604257	0.71369049582822	-0.26036628714947
N	-1.33251677264301	3.40181798243943	0.17676076331700
C	-0.53279800349660	2.36832322533327	-0.03296504087347
N	-1.22107692520732	1.20147244424050	-0.23264012908476
C	0.93455184831632	2.44815309128715	-0.05328238170802
C	1.57692318704039	3.67516340081573	0.16234302503798
C	2.96784926343466	3.71929228933573	0.13588450739438
C	3.68101308432704	2.54261472019652	-0.10238621847741
C	2.96219470786531	1.36514311912076	-0.30570093090351
N	1.62775786886786	1.31807960266072	-0.28286644379240
H	-5.98294143298888	3.22414277634776	0.25874758944275
H	-5.85866564544380	0.79473461008056	-0.19154985058232
H	-3.90943482560144	4.60488837584655	0.46224313481899
H	-3.65446770221938	-0.35696366975784	-0.45938093153067
H	-0.80945852742442	0.27720979651818	-0.40250507843026
H	0.98309007466428	4.56815564596884	0.34580675587077
H	3.49001742447746	4.66302853993874	0.30039586159680
H	4.77026771782267	2.53149029414266	-0.13133882557108
H	3.47805844331392	0.42137675325992	-0.49447363744342
O	0.44018472966192	-1.12281367125278	-0.58873788232859
C	0.49972560028877	-1.77600088563191	0.67435186554055
H	0.97515401880594	-0.28500440944943	-0.53125541774707
H	1.52414207146645	-2.11590008231654	0.89876538111520
H	-0.16436550945591	-2.64989867679033	0.64138888392127
H	0.16319662206847	-1.11091099733287	1.48825822208874

$\underline{S_1 \text{ reactant:}}$

30

E = -741.731725483483 a.u.

C	-5.58462995922319	1.88580310771202	-0.29622906993084
C	-5.27804909497964	0.51671947032238	-0.54926505197211
C	-4.59320120441397	2.82374206674082	-0.07411643923601
C	-3.23755556510606	2.37780817550269	-0.10442199913883
C	-2.95488710417426	0.97433192287445	-0.36698385440273
C	-3.95578364448767	0.04171402495311	-0.59075158491504
N	-2.10112719971306	3.06044252365605	0.06408787152889
C	-1.08545961428291	2.14007854395443	-0.08410492258227
N	-1.59047956358535	0.88049973680725	-0.34038607070228
C	0.29366441561915	2.43817930921032	-0.00087908972266
C	0.72844121438722	3.77445436300227	0.26002765463920
C	2.07929291973693	4.05489207088323	0.32819784271829
C	3.00385041910325	2.99832650470513	0.13513010084158
C	2.50342836603486	1.71163773490381	-0.11960363645181
N	1.21227800532954	1.41000142305416	-0.18953995061105
H	-6.63224389597032	2.19009627520682	-0.28518641433165
H	-6.09864488395654	-0.18173923623604	-0.72097304992169
H	-4.82062806328762	3.87380522320595	0.11184525834053
H	-3.73086115747287	-1.00635905131468	-0.78657255119797
H	-1.02915352558485	0.03000241814516	-0.48435357158684
H	-0.01966986089914	4.55297975177880	0.40053228050837
H	2.42377597202173	5.07162920675862	0.52194548476867
H	4.08048574799738	3.16375781697612	0.17492790819629
H	3.20087302784155	0.88325729189452	-0.27563996110170
O	0.41780731797086	-1.11882315492606	-0.56991846805238
C	0.52440680007874	-1.78955833376424	0.67917405879246
H	0.81727189422456	-0.19861570808842	-0.46877141258541
H	1.57840264547617	-1.98309239070858	0.93921993232637
H	-0.00310871080671	-2.75031693521505	0.60548595012786
H	0.06545430212224	-1.20101415199509	1.49235275565476

S_1 ESPT transition state:

30
E = -741.723549874834 a.u.

C	-3.82453791679878	1.60316091927705	0.35127716746036
C	-3.81718280442300	0.20644166121830	0.07755098362800
C	-2.65226649752808	2.33053793246969	0.42340292105845
C	-1.42235734035326	1.63186041992966	0.21684211947694
C	-1.44547293044945	0.20463509075153	-0.05339381606192
C	-2.62311808413265	-0.51434173550527	-0.12762128775978
N	-0.16454064215797	2.08213669849581	0.22957384034869
C	0.61725940277704	0.97624534867948	-0.02544795658362
N	-0.12728359834395	-0.15315843342956	-0.19597528108178
C	2.04182965135978	0.99972691136424	-0.11424581111135
C	2.77640639998338	2.19184791473511	0.06023897505403
C	4.15989519467146	2.18535455382251	-0.03838151854337
C	4.81821288297059	0.95482345305229	-0.31480172771725
C	4.05405196623202	-0.19270678375775	-0.47732577475410
N	2.71540049264308	-0.19390163118847	-0.38656540154624
H	-4.78388112351561	2.09973799693847	0.50160497655281
H	-4.76931449665387	-0.32357259469410	0.02452340528411
H	-2.64637750062925	3.40135460245876	0.62869875701431
H	-2.63377885225836	-1.58401796989599	-0.33473621225304
H	0.30120312810260	-1.11979793859108	-0.37925473210830
H	2.23129956555829	3.10981901755116	0.27373406302639
H	4.72761859837800	3.10631729802537	0.09229543071690
H	5.90222028802527	0.89462663520705	-0.40546185485796
H	4.51602423834569	-1.15852173641381	-0.68917679854511
O	1.35188933954539	-2.28304775724904	-0.54639622639886
C	1.43754931749597	-2.95782535901807	0.68633281857774
H	2.12487765810965	-1.21747705641482	-0.49473962434836
H	2.13183777564622	-3.81774838173251	0.63848974225725
H	0.45113578534506	-3.34575372041169	1.00380406198655
H	1.79875010205474	-2.29145535567433	1.49690476122855

S_1 ESPT product:

30
E = -741.745400389390 a.u.

C	3.30309422215214	1.65849585023321	-0.23573177920238
H	-5.39372384879492	0.78317179669491	0.09981210004495
H	5.25482729367761	-1.11793207688427	0.24310521168641
C	-3.64301913535288	-0.40009470165700	0.31674740584778
C	-1.48762807364373	0.67737071106195	0.05252036721866
C	4.44057614115789	0.85650972893125	-0.11148448653108
N	-2.27535822239456	-0.45343258760133	0.30502418021358
C	-4.30545358681250	0.76943471006143	0.08514587076463
C	3.09853404556694	-1.14717740392963	0.30314533499521
H	3.38439461862137	2.72722798547798	-0.44234056750001
H	5.42870823766784	1.30750236205907	-0.22224629327772
H	-4.14625013986439	-1.34317463345335	0.51979089782441
H	-1.56247772608256	2.77528073940281	-0.38376837531176
C	-2.16629981146729	1.89136046334199	-0.18677697741725
C	1.96764139748582	-0.34154256630417	0.17838683315842
N	0.61858557486410	-0.61353602868519	0.26124942617601
C	4.34381676211990	-0.52373699028545	0.15329147834468
H	0.20834722637721	-1.52751760471785	0.41402861850154
H	-4.04783636221953	2.90522092588291	-0.36611284528942
H	3.01621773943655	-2.21540486966851	0.50862395519476
C	-0.06480509109058	0.57272893189442	0.04612974662822
C	-3.54255089780355	1.95911892415802	-0.17589881454082
C	2.04350473593645	1.05386044810428	-0.08976628816694
N	0.78109095014580	1.59148783353531	-0.16421438141517
O	-0.98121425153304	-3.06339715996001	0.40379992972536
H	-1.83651365475582	-1.35279642506914	0.47224249444119
C	-1.22874383321990	-3.51100626822922	-0.84348667579837
H	-0.78311391133738	-4.49579799614288	-1.06420814111174
H	-2.34242021458423	-3.64700577729768	-0.90033745088840
H	-1.02353018425276	-2.75521832095386	-1.62717077431477

S$_1$ HAT transition state:

30

E = -741.723565758893 a.u.

C	-5.04787163542672	-1.30405664656799	-1.38471374439138
C	-4.30818501314592	-2.50451238610206	-1.57740734489574
C	-4.43083973677253	-0.13153835367730	-0.99347635472768
C	-3.01682987037359	-0.16132144588719	-0.78426159805194
C	-2.28844397008728	-1.40158332702924	-0.98614044381801
C	-2.91315708687588	-2.56919207882210	-1.38048828040306
N	-2.18158527790748	0.81321512778308	-0.41320776695183
C	-0.93591250545996	0.22471470049821	-0.37246423777075
N	-0.97821294136606	-1.09515536898410	-0.71048826153647
C	0.26710543793785	0.90697997570096	-0.01782630436702
C	0.26986876106930	2.27479402942755	0.32789604459489
C	1.45346880762289	2.90983328105073	0.67460718183213
C	2.65876794363056	2.15358243026501	0.67181377725980
C	2.60794545362233	0.81164715330283	0.32044470494637
N	1.46733680981926	0.19100367897084	-0.01749489756405
H	-6.12489398360081	-1.32183199844944	-1.55593128069227
H	-4.83939570944229	-3.40479091755202	-1.89007290655875
H	-4.98680231431256	0.79499453482749	-0.84716548789564
H	-2.36077452981028	-3.49614197475471	-1.53197268699479
H	-0.11275880215169	-1.72968886030846	-0.71937379583737
H	-0.67575333611561	2.81430288133220	0.31473641622637
H	1.45618922848393	3.96582717876968	0.94357441190259
H	3.61577867295391	2.60286402838654	0.93457785887314
H	3.50749962849428	0.19403785099456	0.30181158228628
O	1.36327599840123	-2.25143008037510	-0.53943849063538
C	1.42982668687008	-2.97204947495407	0.66802727685151
H	1.48899990657007	-0.96712931488826	-0.27390201846891
H	0.70972632476083	-3.81203340876296	0.67670470030852
H	1.19152833779227	-2.33244657565221	1.54305407972992
H	2.43674871481995	-3.39699463854244	0.83948786674954

S$_1$ HAT product:

30
E = -741.741808625278 a.u.

C	-4.10718165995639	3.17181223143573	0.00318320016272
C	-4.14584215053595	1.77044260326456	0.05421668799023
C	-2.87850045778376	3.89794633878272	-0.03990257502528
C	-1.70964231863000	3.17313301328451	-0.02940910595222
C	-1.73733490307223	1.73572652948879	0.02079413452056
C	-2.96585907398298	1.02478177664403	0.06369347538500
N	-0.37566923206149	3.57925578245128	-0.05972769857755
C	0.30439675495994	2.45376628725129	-0.02903688695892
N	-0.47727488770961	1.28529195968903	0.01844227368618
C	1.76235059824602	2.37784943019909	-0.03894363292587
C	2.59187037923838	3.48148602171588	-0.09841458935509
C	3.99084740320493	3.34401377366787	-0.10516099429733
C	4.53519561404935	2.02805297770499	-0.04642878745362
C	3.70036323256348	0.94227064397051	0.01332910294563
N	2.33570351997100	1.10368115079730	0.01411754561514
H	-5.04419279108861	3.72979627792498	-0.00352946079450
H	-5.11009439968768	1.26305276472967	0.08604823256135
H	-2.88023011695593	4.98770814859923	-0.07937792231871
H	-2.97576948088751	-0.06485424936242	0.10287068875577
H	-0.00165979675820	-0.39990693946456	0.10016872788605
H	2.12814192225924	4.46580953196024	-0.14146450416188
H	4.63658588520345	4.21854912302472	-0.15087090532279
H	5.61233236811569	1.86281947307294	-0.04709627007201
H	4.05609751296769	-0.08434402346674	0.06216733516747
O	0.48103480199973	-1.25940554074519	0.21987790146269
C	0.44101544608881	-1.59847390316687	1.60035571345162
H	1.74797979850262	0.27369492209907	0.07404726995389
H	1.04208096677356	-2.50533189748118	1.75095840855986
H	-0.59113610796263	-1.79786869275427	1.93416281178108
H	0.86169117292908	-0.79285551531725	2.22647982333052
